# Development of bacterial sonosensitizer hybrid systems to enhance cancer sono-immunotherapy

**DOI:** 10.1016/j.apsb.2026.03.024

**Published:** 2026-03-17

**Authors:** Haiyan Guo, Yuhan Li, Xue Chen, Xiuru Ji, Zeyang Liu, Hongjing Jiang, Han Wang, Dalong Ni

**Affiliations:** Department of Orthopaedics, Shanghai Key Laboratory for Prevention and Treatment of Bone and Joint Diseases, Shanghai Institute of Traumatology and Orthopaedics, Ruijin Hospital, Shanghai Jiao Tong University School of Medicine, Shanghai 200025, China

**Keywords:** Bacteria, Sonosensitizer, Bacterial sonosensitizer hybrid systems, Live delivery system, Sono-immunotherapy, cGAS–STING pathway, Antigen-presentation, Breast cancer

## Abstract

Microorganisms can activate anti-tumor immune responses *via* the innate immune system. However, this immune effect lacks specificity, and prolonged stimulation by live bacterial colonization may lead to immune tolerance. Sonodynamic therapy triggers cellular death and lysis, fully activating the antigen presentation process by providing heterologous DNA and tumor antigen *in situ*. Herein, to enhance the immunological effect facilitated by ultrasonic treatment, a manganese-containing porphyrin-based metal-organic framework (Mn-MOF) was modified as an acoustic sensitizer on the surface of *Escherichia coli* to form bacterial sonosensitizer hybrid systems (HA@Mn-MOF@E). Importantly, HA@Mn-MOF@E was able to target and colonize 4T1 tumors due to the anoxic tendency of anaerobes. The ultrasound-induced bacterial and tumor cell death and released manganese could activate macrophages and dendritic cells (DCs) through the activation of the cGAS–STING pathway, which increased the proportion of CD3^+^ T cells and M1/M2 ratio within the tumor, as well as CD8^+^ effector T cells and CD86^+^ DCs in lymph nodes. By sono-sensitized immunotherapy, HA@Mn-MOF@E was demonstrated to inhibit orthotopic 4T1 tumor progression and induce tumor necrosis effectively. Such a designed bacterial sonosensitizer hybrid system offered the possibility of using sonodynamic assistance to sensitize live microorganisms-induced immunotherapy, with thorough activation of the antigen presentation in the tumor.

## Graphical abstract

A bacterial sonosensitizer hybrid system was constructed by modification of ECN with Mn-MOF for activating antigen presentation process by combination of sono-sensitized immunotherapy against breast tumor.

**Figure:**
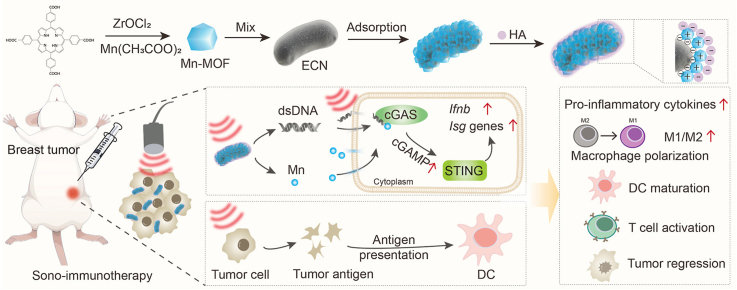

## 1 Introduction

Immunotherapy is a monumental revolution in the treatment of cancer, which rejuvenates the recognition and eradication of tumors by the host immune system^1^. However, most tumors are highly heterogeneous and in an immunosuppressive microenvironment with a low response rate to monotherapy, especially for breast cancer. During treatment, cancer cells express a unique proteome on the cell membrane to acquire new phenotypes to sustain unlimited proliferation, immune escape, and metastasis. Tumor surface antigens are typically diverse and unevenly expressed^2^, ^3^, ^4^, impeding the immune cells’ ability to recognize tumor cells and thus leading to drug resistance. In addition, due to the dense extracellular matrix in breast cancer, most immunotherapeutic agents exhibit low penetration and diffuse poorly within tumors. Therefore, biologic immunotherapies with targeted delivery capabilities, which simultaneously induce *in situ* cleavage of tumor antigens to activate the immunosuppressive microenvironment, enable precise immunotherapy.

Live microorganisms, as an effector that modulates tumor immunity, can sustainably activate the immune response by specifically colonizing and penetrating the tumor compared to chemical activators^5^, ^6^, ^7^. Lipopolysaccharide and flagellin on the surface of bacteria activate Toll-like receptors and initiate a pro-inflammatory response^8^^,^^9^. As heterologous microorganisms, bacteria can induce the activation of innate immunity^10^, ^11^, ^12^ but lack a tumor-specific immune response. Long-term colonization also triggers a chronic inflammatory response, which may lead to depletion of immune cells and ultimately immune tolerance^13^, ^14^, ^15^. Besides, live microbiotherapy could cause side effects such as infections, and the application of inactivated bacterial vectors makes it difficult to exploit anaerobic tropism for delivery to the tumor interior. Hence, adjuvant therapies are often used to superimpose live microbial therapies.

Common adjuvant treatment usually combines live microbial therapy with photothermal therapy^16^^,^^17^, radiotherapy^18^^,^^19^, sonodynamic therapy^20^^,^^21^, etc. Among them, ultrasonic treatment utilizes the mechanical disruption effect and reactive oxygen species (ROS) generated by sonosensitizers to induce the immunogenic cell death (ICD), providing antigens for the anti-tumor immune response^22^^,^^23^. Under low-intensity ultrasound, porphyrin, as a sonosensitive agent, can produce ROS to induce lipid peroxidation of cell membranes and ultimately lead to mitochondrial damage and cell fragmentation^24^, ^25^, ^26^. Most sonodynamic studies have been focused on anti-microbial therapy^27^, ^28^, ^29^ or simply using bacteria as acoustic sensitizer delivery vehicles^30^^,^^31^. However, the integration of sonodynamic-sensitized live bacterial immunotherapy with a single material remains challenging. The materials must simultaneously respond to both sonodynamic and immunotherapeutic stimuli, promoting the fragmentation of tumor cells and bacteria into immunostimulatory debris. The immunogenic substances of microorganisms, such as double-stranded DNA (dsDNA), stimulate the activation of the cyclic guanosine monophosphate-adenosine monophosphate synthase-stimulator of interferon genes (cGAS–STING) pathway^32^, ^33^, ^34^. Meanwhile, sonosensitive porphyrins can chelate Mn^2+^ ions, which have been reported to activate cGAS to enhance its recognition of dsDNA and thus enhance the activation of the STING pathway^35^. In addition, the *in situ* ultrasonic effect promotes the fragmentation of tumor cells, leading to the release of antigens and facilitating the process of tumor antigen presentation. The immunostimulatory effects induced by ultrasound-mediated release of Mn^2+^ ions, exogenous bacterial DNA, and cellular antigens may serve as potent immunotherapeutic agents against breast cancer.

**Figure 1:**
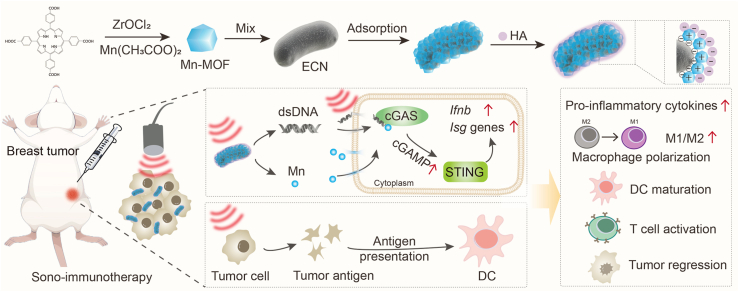
Schematic diagram of a bacterial sonosensitizer hybrid system, which was constructed by modification of ECN with Mn-MOF for activating the antigen presentation process *via* sonosensitized immunotherapy against breast tumors.

Herein, a bacterial sonosensitizer hybrid system (HA@Mn-MOF@E) was constructed by modifying the Mn-porphyrin organometallic framework (Mn-MOF) as a sonosensitizer onto the surface of live bacteria named *Escherichia coli* Nissle 1917 (ECN) (Fig. 1). Relying on the hypoxia-tropic ability of bacteria and the tumor hypoxic microenvironment, the HA@Mn-MOF@E system optimized the efficient delivery and distribution of therapeutic agents within tumors. Ultrasound was then used to activate the sonosensitizer to promote the lysis of bacterial and tumor cells. Simultaneously, ultrasound induced transient pores in the cell membrane, facilitating the entry of dsDNA and Mn^2+^ ions into the cytoplasm. Subsequently, the released bacterial contents and Mn^2+^ ions jointly activated the cGAS–STING pathway, prompting the presentation of the released tumor antigens to enhance the antitumor immune response, which suppressed *in situ* tumor progression. The developed hybrid bacterial system provides a new paradigm for sonosensitized immunotherapy derived from living microorganisms and a promising compositional model for ultrasound-enhanced tumor immunotherapy.

## 2 Materials and methods

### 2.1 Reagents

Meso-tetra(4-carboxyphenyl) porphine (TCPP) was obtained from Macklin. The 5-ethynyl-2′-deoxyuridine (EDU) and Azide-AF488 Kit, SOSG Assay Kit, Fluo-4 AM, and CCK-8 were purchased from Beyotime Biotechnology. Colony-stimulating factor (CSF) and interleukin-4 (IL-4) were purchased from Thermo Fisher. Antibodies for Western blot, including phospho-TBK1 (p-TBK1) rabbit antibody, phospho-IRF3 (p-IRF3) rabbit antibody, phospho-P65 (p-P65) rabbit antibody, TBK1 rabbit antibody, IRF3 rabbit antibody, P65 rabbit antibody, vinculin antibody, and anti-rabbit IgG-HRP, were purchased from Cell Signaling Technology. Antibodies for flow cytometry, including PE-Cy7-CD86, FITC-CD206, PE-CD80, PE-F4/80, FITC-CD11c, APC-CD45, APC-Cy7-CD3, FITC-CD4, PE-Cy7-IFN-*γ* and PerCP-Cy5.5-CD11b were purchased from Biolegend. Luria–Bertani (LB) broth and LB agar were purchased from Sangon. Dulbecco's modified Eagle's medium (DMEM) was obtained from Gibco. The rest of the chemical reagents were of analytical grade.

### 2.2 Studies in animals

4T1 cells and RAW264.7 (from ATCC) were cultured with DMEM containing 10% FBS and 1% penicillin–streptomycin in a 37 °C culture incubator. ECN was cultured with LB broth or LB agar plates in a 37 °C bacterial incubator.

SPF-grade female BALB/c and C57BL/6 mice were provided by Shanghai SLAC Laboratory Animals Co., Ltd. All animal experiments were approved by the Laboratory Animal Ethics Committee of Shanghai Jiao Tong University (A2024339) and were conducted in compliance with the National Research Council's Guide for the Care and Use of Laboratory Animals under standard breeding environments in the Laboratory Animal Center of Shanghai Jiao Tong University.

### 2.3 Preparation of Mn-MOF

To prepare Mn-MOF, 300 mg of ZrOCl_2_·8H_2_O, 2800 mg of benzoic acid, and 100 mg of TCPP were dissolved in 100 mL of *N*,*N*-dimethylformamide (DMF). The mixture was heated at 90 °C for 5 h. Then it was washed three times. Subsequently, 2.5 mmol of manganese acetate dissolved in DMF was added, and the mixture was stirred at 70 °C for 5 h, then washed with DMF three times and stored at 4 °C.

### 2.4 Preparation of HA@Mn-MOF@E

To prepare HA@Mn-MOF@E, 155 nmol of Mn-MOF was washed once with double-distilled water (DDW), and then resuspended in 75 μL of DDW. ECN was cultured overnight, and 0.6 mL of the culture was washed once with DDW. Then the bacteria were mixed with 155 nmol of the Mn-MOF, and the mixture was shaken at 1500 rpm (MVM-2500, Titan, Shanghai, China) for 15 min. Subsequently, 200 μL of hyaluronic acid (HA) (10 mg/mL) was added and shaken for 5 min. The mixture was washed to obtain HA@Mn-MOF@E.

### 2.5 Characterization of HA@Mn-MOF@E

To prepare the TEM sample, 10^7^ CFU of HA@Mn-MOF@E was dropped onto a copper grid and then washed twice. The morphology was observed by transmission electron microscopy (TEM), and the content of elements such as N, Zr, and Mn on the surface was analyzed using energy-dispersive X-ray spectroscopy (EDS). Meanwhile, the freeze-dried powder of HA@Mn-MOF@E was analyzed for the elemental composition using X-ray photoelectron spectroscopy (XPS) (Thermo Kalpha). Since Mn-MOF can be excited to red fluorescence, HA@Mn-MOF@E was prepared using GFP-expressing ECN. Then HA@Mn-MOF@E was dropped onto an agar thin film and observed under a confocal microscope. The fluorescence intensity of ECN and HA@Mn-MOF@E in the PE fluorescence band was measured using flow cytometry (Beckman). A Zetasizer (Malvern) was utilized to measure the size and potential changes of the bacteria before and after modification. The content of manganese was determined by inductively coupled plasma optical emission spectrometry. HA@Mn-MOF@E was diluted 10^5^ times, spread on a plate, incubated at 37 °C for 24 h, and the number of colonies was counted to assess the bacterial viability. Furthermore, to evaluate the effect of modification on bacterial growth, ECN and HA@Mn-MOF@E were added to LB medium at room temperature, and the OD 600 values were continuously monitored using a microplate reader.

### 2.6 *In vitro* anti-tumor effect induced by ultrasound

To verify the cell-killing effect by MOFs under sonication, approximately 10^7^ CFU of ECN and HA@Mn-MOF@E was treated with 1.5 W/cm^2^ of ultrasound for 5 min. Then, the bacteria were spread on plates to be counted and photographed. Then, ultrasound-induced cellular uptake of dsDNA and Mn^2+^ was verified on 4T1 cells. First, EDU was added to the LB medium and ECN was incubated for 4 h. EDU-labeled ECN was used to prepare HA@Mn-MOF@E, which was then sonicated at 1.5 W/cm^2^ for 5 min. The supernatant was collected by centrifugation, yielding the sonication-released EDU-dsDNA. The released EDU-dsDNA was co-incubated with 4T1 cells and sonicated at 1.5 W/cm^2^ for 10 s. Subsequently, cells were stained with azide-AF488 according to the kit protocol, and the fluorescence was visualized by confocal microscopy. To investigate the form of manganese ion entry into cells, 4T1 cells were co-incubated with sonicated HA@Mn-MOF@E and non-sonicated HA@Mn-MOF@E for 1 h, followed by observation of Fluo-4 AM staining for divalent ions inside the cells.

Besides, their cytotoxic effects on tumor cells were investigated. 1 × 10^4^ 4T1 cells were inoculated and treated with PBS, US, ECN, HA@Mn-MOF, HA@Mn-MOF + US, and HA@Mn-MOF@E + US, respectively. The ultrasound power was 1.5 W/cm^2^ for 5 min. After 2 h of incubation, cells were washed to remove bacteria. DMEM complete medium containing CCK-8 was then added, and the absorbance value at 450 nm was measured using a microplate reader. To further evaluate the ability to induce apoptosis *in vitro*, 4T1 cells were treated with PBS, HA@Mn-MOF, ECN, and HA@Mn-MOF@E at the same bacterial dosage. The ultrasound group was treated with 1.0 W/cm^2^ ultrasound for 3 min, and then cultured for 2 h in a 37 °C incubator. Cells were stained with Annexin V and PI and analyzed using flow cytometry. Finally, the cytotoxicity ability against 4T1 cells was assessed using a visual live-dead cell staining method. The same treatment as described above was performed with 1.0 W/cm^2^ ultrasound. After 2 h of incubation, the cells were washed three times with PBS, and then calcein-AM and PI were added, and the cells were stained. The singlet oxygen induced by sonication was detected using the SOSG Assay Kit with a microplate reader. To evaluate the interaction of HA@Mn-MOF@E with cells, equal amounts of HA@Mn-MOF and HA@Mn-MOF@E were incubated with 4T1 cells for 1 h, and the red fluorescence of MOF on the cell surface was observed under confocal microscopy.

### 2.7 *In vitro* immune activation effect

To verify the activating effect of HA@Mn-MOF@E on immune cells *in vitro*, two types of antigen-presenting cells, macrophages and DCs, were selected for the experiment. First, HA@Mn-MOF and HA@Mn-MOF@E were treated with 1.5 W/cm^2^ of US for 5 min, respectively. Then, 1 × 10^6^ RAW264.7 cells were co-cultured with PBS, ECN, HA@Mn-MOF, HA@Mn-MOF + US, and HA@Mn-MOF@E + US. Flow cytometry was used to determine CD86^+^ cells. To further validate the immune activation, RAW264.7 cells were co-cultured with PBS, ECN, HA@Mn-MOF, HA@Mn-MOF + US, and HA@Mn-MOF@E + US, and then cultured at 37 °C in an incubator for 12 h. Then, RNA was extracted with Trizol reagent, and polymerase chain reaction (PCR) was used to detect inflammatory genes such as *Tnfa*, *Il6*, *Ifnb*, and *Isg*. The cyclic guanosine monophosphate–adenosine monophosphate (cGAMP) was extracted after 4 h incubation using a mixture of methanol, acetonitrile, and water in a 4:4:2 ratio. The sample was dissolved in a 10 mmol/L ammonium acetate solution and analyzed by HPLC–MS (Waters). Water and acetonitrile, both containing 0.1% formic acid, were used as mobile phases. Detection was conducted at multiple reaction monitoring mode and 1 kV of capillary voltage (ESI positive mode). To validate the expression of p-TBK1, p-IRF3, and p-P65, the RAW264.7 cells were co-incubated with the supernatant of PBS, ECN, HA@Mn-MOF, sonicated HA@Mn-MOF, and sonicated HA@Mn-MOF@E for 12 h. The obtained cell lysate was loaded onto a 4%–12% gradient gel at 15 μg/well, followed by electrophoresis and subsequent membrane transfer. P-TBK1 rabbit antibody, p-IRF3 rabbit antibody, p-P65 rabbit antibody, TBK1 rabbit antibody, IRF3 rabbit antibody, P65 rabbit antibody, vinculin antibody, and anti-rabbit IgG-HRP were used for Western blot.

In addition, bone marrow cells were also extracted from 6-week-old C57BL/6 mice and stimulated with CSF and IL-4 to form mature DCs. Then, DCs were co-incubated with PBS, ECN, HA@Mn-MOF, sonicated HA@Mn-MOF, and sonicated HA@Mn-MOF@E (1.5 W/cm^2^ ultrasound for 5 min), respectively. Flow cytometry was used to analyze the proportion of CD80^+^ and CD86^+^ DCs. Furthermore, to validate the ICD effect, 4T1 cells were treated with PBS, US, ECN, HA@Mn-MOF, HA@Mn-MOF + US and HA@Mn-MOF@E + US, respectively. Then the supernatant was incubated with DCs. Flow cytometry was used to analyze the proportion of CD86^+^ DCs.

### 2.8 Biodistribution

To validate the retention effect of HA@Mn-MOF@E following intratumoral injection, 1 × 10^6^ 4T1 cells were inoculated into the right mammary gland of 6-week-old female BALB/c mice. When the tumor size reached 100 mm^3^, 5 × 10^5^ CFU of ECN, HA@Mn-MOF@E, and HA@Mn-MOF with the same fluorescence intensity were injected into tumors. The fluorescence signals of 633 nm wavelength were monitored for 72 h. At 72 h after injection, tumors, hearts, livers, spleens, lungs, and kidneys were collected. The fluorescence signals of luciferase bioluminescence in isolated tissues were detected and quantified using an *in vivo* imaging system. Meanwhile, blood and various tissues and organs were collected, weighed, and homogenized. The number of ECN was counted by the bacterial smear method.

To further verify the tumor-targeting and colonization ability of HA@Mn-MOF@E, 1 × 10^7^ 4T1 cells were inoculated subcutaneously into female BALB/c mice to establish a subcutaneous breast cancer tumor model. When the tumor size reached 100 mm^3^, 5 × 10^5^ CFU of ECN, HA@Mn-MOF@E, and HA@Mn-MOF with the same fluorescence intensity were injected intravenously. At 72 h after injection, tumors, hearts, livers, spleens, lungs, and kidneys were collected. The fluorescence signals of luciferase bioluminescence and 633 nm wavelength in isolated tissues were detected and quantified using an *in vivo* imaging system, respectively. Meanwhile, blood and various tissues and organs were collected, weighed, and homogenized. And ECN colonies were counted by the plate count method.

### 2.9 *In vivo* immune effect analysis

To analyze the *in vivo* immune effects, PBS, HA@Mn-MOF, 5 × 10^5^ CFU of ECN, and HA@Mn-MOF@E were injected into BALB/c mice bearing 4T1 subcutaneous tumors. Two hours after administration, the ultrasound-treated group received 1.5 W/cm^2^ ultrasound irradiation for 5 min. At 72 h post ultrasound treatment, tumor tissue and lymph nodes were collected to form a single-cell suspension. Then, the ratio of CD3^+^ T cells, IFN-γ^+^CD8^+^ T cells, CD86^+^/CD206^+^ macrophages, and CD86^+^ macrophages was analyzed by flow cytometry. Subsequently, 20 mg of tumor tissue was taken, and the expression levels of *Tnfa*, *Cd206*, *Il6*, and STING pathway-related genes *Ifnb*, *Isg*, and *Ccl5* were determined by PCR. Meanwhile, paraffin sections of tumor tissues were prepared and stained for CD3 and CD8 lymphocytes on Day 4 after drug administration.

### 2.10 *In vivo* anti-tumor assay

An orthotopic breast tumor was established by inoculating 1 × 10^6^ 4T1 cells into the right mammary gland of 6-week-old female BALB/c mice to verify the anti-tumor effect of HA@Mn-MOF@E. When the tumor grew to nearly 100 mm^3^, PBS, HA@Mn-MOF, 5 × 10^5^ CFU of ECN, or HA@Mn-MOF@E were injected into the tumor. At 2 h and 24 h after injection, 5-min 1.5 W/cm^2^ ultrasound treatments were performed with ultrasound instrumentation on the HA@Mn-MOF + US group and the HA@Mn-MOF@E + US group. The day of administration was recorded as Day 0. The tumor volume was calculated using Eq. (1):

(1) $$V=\text{Length}\times {\text{Width}}^{2}\;/\;2$$

Meanwhile, the body weights of the mice were monitored. On Day 12, tumor tissues were collected, photographed, and weighed. Mice receiving the aforementioned intratumoral injection were continuously monitored for up to 48 days. On Day 40, 1 × 10^6^ 4T1 cells were inoculated into the contralateral breast to rechallenge. To record the survival time, mice with tumor size exceeding 2000 mm^3^ were euthanized immediately. Tumor tissues were also collected on Day 3 after ultrasound treatment to detect the pathological changes, paraffin-embedded, and the sections were stained with H&E, TUNEL, and Ki67. To investigate the pharmacodynamics of intravenous administration, an orthotopic breast tumor was established by inoculating 1 × 10^6^ 4T1 cells into the right mammary gland of 6-week-old female BALB/c mice. When the tumor grew to nearly 100 mm^3^, PBS, HA@Mn-MOF, 5 × 10^5^ CFU of ECN, or HA@Mn-MOF@E were administered intravenously. At 24 h and 48 h post-injection, a 5-min 1.5 W/cm^2^ ultrasound treatment was performed on the HA@Mn-MOF + US group and the HA@Mn-MOF@E + US group. The day of administration was recorded as Day 0. On Day 12, all mice were photographed. Mice were euthanized when tumor size exceeded 2000 mm^3^. The tumors were monitored continuously to obtain the survival curves.

### 2.11 Statistical analysis

Statistical analysis was performed using GraphPad Prism, and data are presented as mean ± standard deviation (SD). Unpaired Student's *t*-test, one-way analysis of variance (ANOVA), and Log-rank test were used in statistical significance analysis. ∗*P* < 0.05, ∗∗*P* < 0.01, ∗∗∗*P* < 0.001, and ∗∗∗∗*P* < 0.0001 represent different statistical significances. ns represents not significant.

## 3 Results and discussion

### 3.1 Preparation and characterization of a hybrid bacterial system

**Figure 2:**
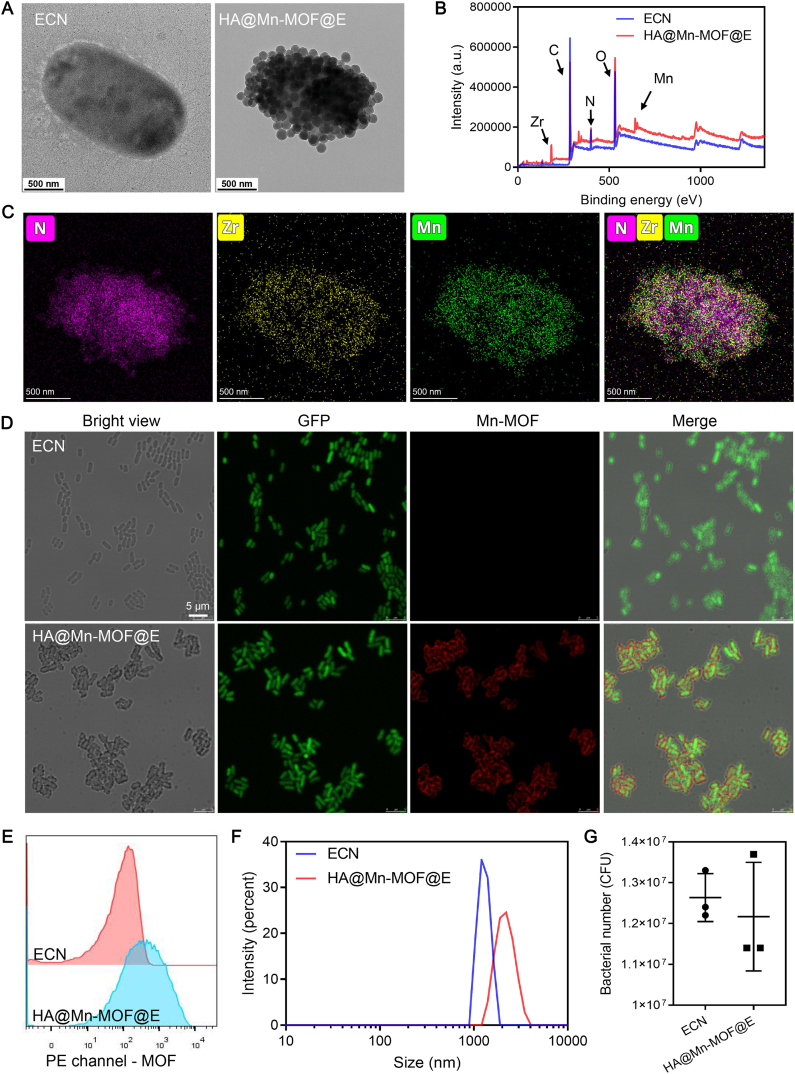
Characterization of HA@Mn-MOF@E. (A) TEM images of ECN and HA@Mn-MOF@E, scale bar = 500 nm; (B) XPS spectra of ECN and HA@Mn-MOF@E; (C) TEM-EDS elemental mapping of HA@Mn-MOF@E; (D) confocal microscopy images of GFP-expressing ECN and HA@Mn-MOF@E, scale bar = 5 μm; (E) representative flow cytometry plots of ECN and HA@Mn-MOF@E; (F) particle size of ECN and HA@Mn-MOF@E; (G) viability of bacteria after modification as determined by plate counting (*n* = 3). Data are presented as mean ± SD.

To simultaneously modify the acoustic-sensitive material porphyrin and the immune activator Mn, bimetallic MOF nanoparticles were synthesized using a hydrothermal method to enable porphyrin and benzoic acid to chelate Zr and Mn (Supporting Information Fig. S1). Microbial surface engineering modifications with MOF are typically realized based on biomineralization and charge interactions^36^^,^^37^. Since Mn-MOF was positively charged, MOF could be modified onto the negatively charged probiotic surface by a simple mixing method, and then wrapped with HA to balance the charge to obtain the HA@Mn-MOF@E. TEM in Fig. 2A shows that the Mn-MOF was successfully coated on the surface of the bacteria. XPS and TEM elemental distribution energy spectra (Fig. 2B and C) also confirmed the changes in the elemental composition of the surface modification. Zr and Mn elements appeared on the surface of the bacteria after modification. Meanwhile, the MOF with red fluorescence was evenly coated on the surface of the bacteria by confocal laser scanning microscopy and flow cytometry analysis (Fig. 2D and E). The average particle size of the bacteria (1369 ± 58 nm) increased to 2155 ± 117 nm after modification (Fig. 2F), while the zeta potential of HA@Mn-MOF@E reduced slightly due to HA coating (Supporting Information Fig. S2). 1 × 10^6^ CFU of ECN was modified with approximately 3.9 ± 0.1 μg of Mn. Plate counting results showed that the survival rate of HA@Mn-MOF@E was similar to that of naked bacteria (Fig. 2G), and the modification exhibited a negligible effect during the exponential growth phase (Supporting Information Fig. S3), indicating that the preparation process of modified acoustic-sensitive materials was highly biocompatible. The above results demonstrated the successful construction of the bacterial sonosensitizer hybrid system.

### 3.2 *In vitro* cytotoxicity induced by ultrasound

**Figure 3:**
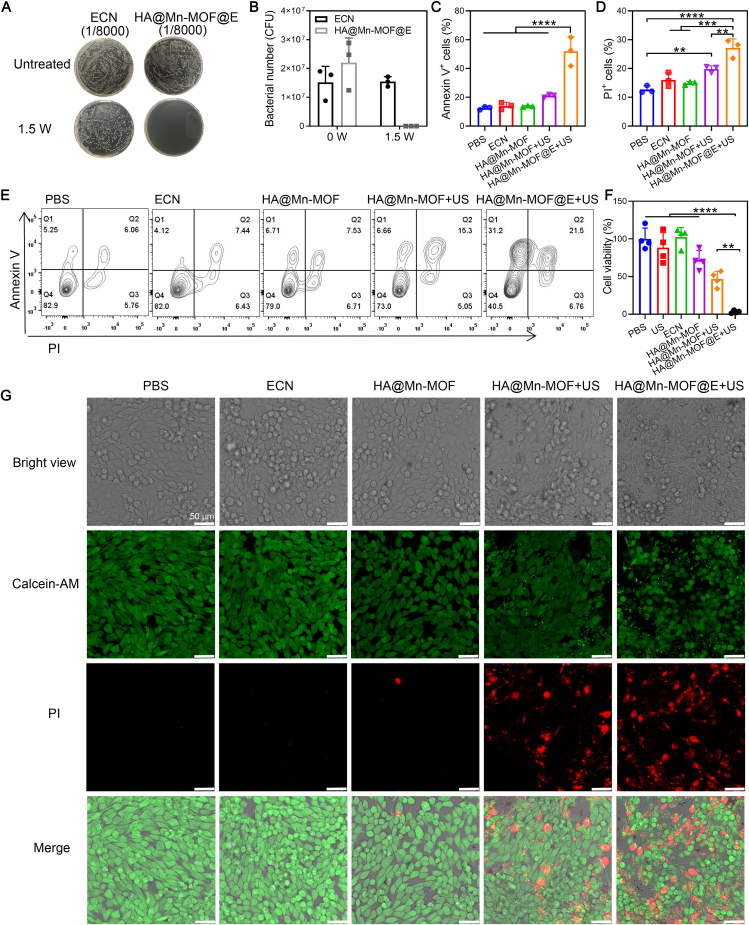
*In vitro* cytotoxicity of HA@Mn-MOF@E triggered by ultrasound. (A) Plate photos after treating HA@Mn-MOF@E with 1.5 W/cm^2^ ultrasound for 5 min and (B) statistical data (*n* = 3); (C, D) quantitative analysis and (E) representative flow cytometry plots of cell apoptosis with staining of APC-annexin V and PI after treatment with PBS, ECN, HA@Mn-MOF, HA@Mn-MOF + US or HA@Mn-MOF@E + US (*n* = 3); (F) cell viability of 4T1 detected using CCK-8 (*n* = 4); (G) confocal microscopy images of 4T1 cells for live and dead staining. Scale bar = 50 μm. Data are presented as mean ± SD. Statistical analysis was carried out by means of one-way ANOVA. ∗∗*P* < 0.01; ∗∗∗*P* < 0.001; ∗∗∗∗*P* < 0.0001.

As a living acoustic sensitizer, HA@Mn-MOF@E was expected to destroy tumor cells under ultrasound. Since MOFs were modified on the bacterial surface, the bacteria died first after 5 min of treatment with 1.5 W/cm^2^ of ultrasound stimulation (Fig. 3A and B), thus promoting better interaction of the fragmented bacteria and their contents with immune cells. Then, the released dsDNA was incubated with 4T1 cells and subjected to ultrasonic treatment for 10 s, followed by azide-AF488 staining (Supporting Information Fig. S4A). Only the ultrasonically treated group exhibited strong green fluorescence, indicating that the cells had engulfed EDU-labeled dsDNA via transient pores induced by sonication. To verify the form of Mn entering cells after ultrasonic treatment, Fluo-4 AM reagent was employed to monitor intracellular Mn^2+^ changes during a 1-h co-incubation of cells with sonicated HA@Mn-MOF@E. The cellular ion staining image (Fig. S4B) indicated that ultrasonic treatment promoted the release of Mn^2+^, enabling its uptake by cells in ionic form. In addition, PBS, HA@Mn-MOF, ECN, and HA@Mn-MOF@E containing the same number of bacteria were co-cultured with 4T1 cells for 2 h, and then HA@Mn-MOF and HA@Mn-MOF@E were treated with ultrasound at 1.5 W/cm^2^. Both early-stage apoptotic cells (Annexin V^+^ PI^–^) and late-stage apoptotic cells (Annexin V^+^ PI^+^) were significantly increased in the HA@Mn-MOF@E group compared with the other groups (Fig. 3C–E). Specifically, the proportion of early apoptotic cells increased to 31.2% in the HA@Mn-MOF@E + US group, whereas it was only 6.66% in the HA@Mn-MOF + US group. This indicates that the acoustic sensitization of HA@Mn-MOF@E can exacerbate tumor cell apoptosis. After 4 h of post-treatment incubation, cell viability was determined by CCK-8, and the results in Fig. 3F and Supporting Information Fig. S5 showed that there was a negligible impact on cell survival in the ultrasound-alone group and the ECN group, while almost all the cells died in the treatment with 1 × 10^6^ CFU/mL of HA@Mn-MOF@E after sonication. However, about 50% of the cells in the HA@Mn-MOF sonication group still survived, which may be attributed to the fact that HA@Mn-MOF, without bacteria, was dispersed in the solution, while HA@Mn-MOF@E was deposited on the cell surface. To validate this assumption, 4T1 cells were observed by confocal microscopy after co-incubation with HA@Mn-MOF@E for 2 h. It was shown that more HA@Mn-MOF@E was deposited on the surface of the cells due to bacterial sedimentation (Supporting Information Fig. S6), whereas the HA@Mn-MOF tended to be dispersed, so the disparity in the sonication effect may be caused by the different distribution of the Mn-MOF on the cell surface. Additionally, the cytotoxic effects may also be attributed to ultrasound-triggered singlet oxygen generation (Supporting Information Fig. S7). The results of live-dead cell staining in Fig. 3G also showed that there were a large number of PI^+^ cells in the US-treated group, and cell debris appeared in the HA@Mn-MOF@E group. The above results indicated that under ultrasound, HA@Mn-MOF@E was able to trigger the death of bacteria and apoptosis of 4T1 tumor cells, leading to the generation of cellular debris.

### 3.3 *In vitro* activation of antigen-presenting cells

**Figure 4:**
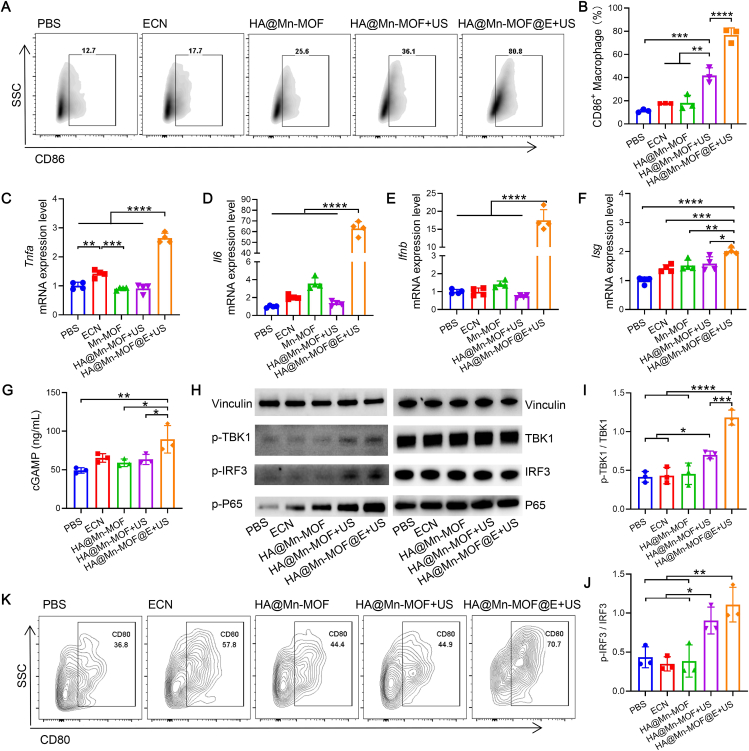
*In vitro* immune activation effect. (A) Representative flow cytometry plots and (B) quantitative analysis of Raw264.7 cells after treatment with PBS, ECN, HA@Mn-MOF, HA@Mn-MOF + US, and HA@Mn-MOF@E + US for 4 h (*n* = 3). The relative gene expression levels of (C) *Tnfa*, (D) *Il6*, (E) *Ifnb*, and (F) *Isg* in Raw264.7 cells after 12 h of co-incubation (*n* = 4). (G) cGAMP levels in macrophages after 4 h co-incubation with each group (*n* = 3). (H) Representative and quantitative data showing (I) p-TBK1, (J) p-IRF3 protein levels in macrophages after 12 h of co-incubation with each group (*n* = 3). (K) Representative flow cytometry plots of CD80^+^ DCs after treatment with PBS, ECN, HA@Mn-MOF, HA@Mn-MOF + US, and HA@Mn-MOF@E + US. Data are presented as mean ± SD. Statistical analysis was carried out by means of one-way ANOVA. ∗*P* < 0.05; ∗∗*P* < 0.01; ∗∗∗*P* < 0.001; ∗∗∗∗*P* < 0.0001.

Tumor immune tolerance occurs mainly due to the difficulty in recognition of tumor antigens by antigen-presenting cells, exacerbated by immunosuppression that prevents the initiation of an antitumor immune response. Activation of macrophages and dendritic cells is critical for promoting antigen presentation. Mn^2+^ ions are metal ions with immune-activating effects, which activate the cGAS–STING pathway by binding and activating cGAS that recognizes dsDNA^35^^,^^38^. To verify the immune activation effect of HA@Mn-MOF@E, macrophages and DCs were examined *in vitro*. Specifically, PBS, HA@Mn-MOF, ECN, sonicated HA@Mn-MOF, and sonicated HA@Mn-MOF@E were co-cultured with Raw264.7 cells for 4 h at the same dose of active ingredients. After ultrasound treatment, about 80% of the macrophages in the HA@Mn-MOF@E group were activated to CD86-expressing M1-type (Fig. 4A and B, Supporting Information Fig. S8A), whereas only about 20% and 40% could be activated in the ECN and HA@Mn-MOF + US groups, respectively. Sonicated HA@Mn-MOF@E increased the percentage of macrophage activation, probably due to the release of dsDNA and Mn^2+^ ion triggered by sonication. The relative gene expression of inflammatory factors was also determined after 12 h of co-incubation of HA@Mn-MOF@E with macrophages (Fig. 4C–E), where increased expression of *Tnfa*, *Il6*, and *Ifnb* inflammatory factors was found. Moreover, compared to the ECN and HA@Mn-MOF groups, the HA@Mn-MOF@E + US group exhibited significantly elevated intracellular cGAMP concentrations, along with increased expression levels of STING pathway-related *Isg* genes, p-TBK1, and p-IRF3, indicating that the combination of bacteria and Mn enhanced the activation of the STING pathway (Fig. 4F–J). Additionally, the expression of p-P65, a marker of the NF-*κ*B pathway, was correspondingly increased in the HA@Mn-MOF@E + US group (Fig. 4H and Fig. S9). Meanwhile, PBS, ECN, HA@Mn-MOF, sonicated HA@Mn-MOF, and sonicated HA@Mn-MOF@E were co-incubated with DCs for 12 h. As shown in Fig. 4K and Supporting Information Figs. S8B and S10, it was found that sonicated HA@Mn-MOF@E increased the proportion of CD80^+^ DCs and CD86^+^ DCs compared with other groups, which indicated that the antigen presentation pathway was activated in DCs by HA@Mn-MOF@E. Furthermore, the supernatant of 4T1 cells treated with PBS, US, ECN, HA@Mn-MOF, HA@Mn-MOF + US, and HA@Mn-MOF@E + US was respectively incubated with DCs to validate the effect of ICD on immune activation. As shown in Supporting Information Fig. S11, ultrasonically induced tumor cell death could activate about 15.6% of DCs. In the HA@Mn-MOF@E + US group, the proportion of activated DCs significantly increased, reaching approximately 2-fold that of the ultrasound-only group. Thus, by virtue of Mn^2+^, heterologous microbial debris and ICD triggered by sonication, HA@Mn-MOF@E was able to activate macrophages and DCs through the stimulation of the STING pathway and increase the expression of inflammatory factors, which enhanced antigen delivery efficiency.

### 3.4 Tumor-targeting colonization

**Figure 5:**
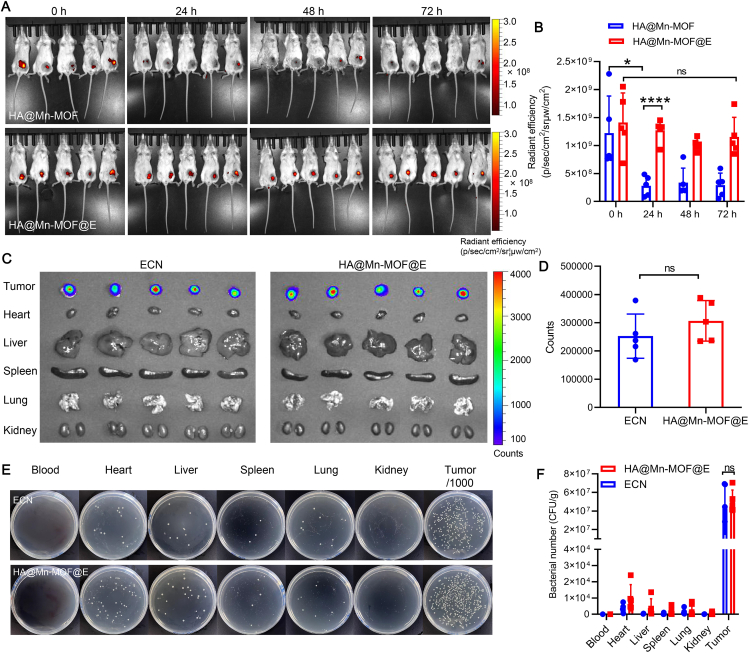
Tumor retention and colonization ability of bacterial sonosensitizer hybrid systems after intratumoral injection. Luciferase-expressing ECN was modified with Mn-MOF and HA@Mn-MOF@E was then administered via intratumoral injection at a dose of 5 × 10^5^ CFU to mice bearing 4T1 orthotopic tumors. Meanwhile, the same amount of HA@Mn-MOF was injected into tumors. (A) Fluorescence imaging (633 nm) from porphyrins in MOF during 72 h and (B) quantitative statistical results (*n* = 5). (C) Bioluminescence imaging of major tissues at 72 h and (D) quantitative statistical results (*n* = 5). (E) Plate images and (F) quantitative statistical results of bacterial number in blood and major tissues (*n* = 5) at 72 h after intratumoral injection of ECN and HA@Mn-MOF@E. Data are presented as mean ± SD. Statistical analysis was carried out using an unpaired Student's *t*-test. ∗*P* < 0.05; ∗∗∗∗*P* < 0.0001; ns represents not significant.

The accumulation and dispersion of nanomaterials within the tumor has a crucial impact on ultrasound-mediated tumor elimination. It has been reported that ECN can colonize the anaerobic environment of tumors, endowing carried nanomaterials to be uniformly distributed within the tumor area^39^. The distribution characteristics of HA@Mn-MOF@E after intratumoral injection were first validated. About 5 × 10^5^ CFU of HA@Mn-MOF@E and an equivalent amount of HA@Mn-MOF were injected into the *in situ* 4T1 mammary tumors of BALB/c mice. In the HA@Mn-MOF group, the red fluorescence intensity of Mn-MOF within the tumor significantly decreased within 24 h (Fig. 5A and B). In contrast, the fluorescence intensity of the HA@Mn-MOF@E group remained stable within the tumor, showing no significant attenuation even at 72 h, thereby extending the therapeutic window for sonodynamic therapy. In addition, equal doses of luciferase-labeled ECN and HA@Mn-MOF@E were injected into tumors, and the blood, tumors, and major organs were collected after 72 h. As shown in Fig. 5C and D, the luciferase signals in tumors between the two groups were comparable and exclusively detected in tumor tissues. Furthermore, bacterial counts in tumors from both groups showed no significant difference, while being markedly higher than those in other major organs (Fig. 5E and F). This indicated that there was no significant difference in tumor colonization ability between HA@Mn-MOF@E and bare bacteria following intratumoral injection.

To further verify the tumor-targeting and colonization ability of ECN with surface modification of HA@Mn-MOF (HA@Mn-MOF@E), 4T1 cells were inoculated into BALB/c mice to establish a subcutaneous breast tumor. To ensure *in vivo* safety, HA@Mn-MOF@E was co-incubated with mouse blood, and it was found that the modification of HA@Mn-MOF did not increase ECN-induced hemolysis, which suggested it was blood-compatible (Supporting Information Fig. S12). Using an *in vivo* imaging system, tumors and major organs were collected 72 h after intravenous injection of 5 × 10^5^ CFU of ECN or HA@Mn-MOF@E to image luciferase fluorescence expressed by ECN. In Supporting Information Fig. S13A and S13B, it was shown that the fluorescence of luciferase appeared only within the tumor, indicating that ECN was able to colonize the tumor. Moreover, there was no significant difference in the fluorescence intensity between the HA@Mn-MOF@E group and the ECN group, implying that the modification with Mn-MOF had no significant effect on its targeting ability towards the anaerobic tumor microenvironment. Meanwhile, monitoring of MOF fluorescence within 72 h (Fig. S13C and S13D) showed that the fluorescence value of HA@Mn-MOF@E within the tumor was significantly higher than that of the HA@Mn-MOF group, suggesting that Mn-MOF could be carried by ECN for targeted delivery into the tumor. Finally, 5 × 10^5^ CFU of HA@Mn-MOF@E and ECN were injected intravenously in subcutaneous tumor-bearing mice. Bacterial counts in various tissues and organs after 72 h showed that the bacterial number in the tumors was higher than that in the blood and in other organs (Fig. S13E and S13F), suggesting that HA@Mn-MOF@E could specifically colonize the tumors. The ECN and HA@Mn-MOF@E groups had similar numbers of bacteria in the tumor, demonstrating that the surface engineering had a negligible impact on the delivery of bacteria^40^^,^^41^. Therefore, HA@Mn-MOF@E exhibited excellent tumor-targeting and colonization ability as ECN.

### 3.5 *In vivo* immune activation

**Figure 6:**
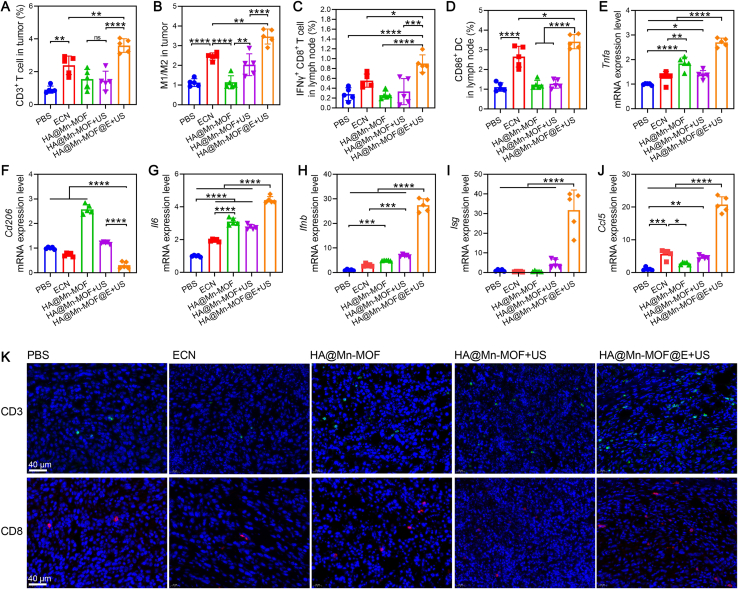
Immune activation within the tumor. At 72 h post injection, (A) the proportion of CD3^+^ cells and (B) the M1/M2 ratio in the tumors (*n* = 5), (C) the proportion of IFN-*γ*^+^CD8^+^ T cells and (D) the proportion of CD86^+^ DCs in the lymph nodes (*n* = 5), the tumor relative gene expression levels of inflammatory factors (E) *Tnfa*, (F) *Cd206*, (G) *Il6*, and STING pathway-related genes including (H) *Ifnb*, (I) *Isg*, and (J) *Ccl5* were determined (*n* = 5). (K) Immunofluorescence staining of tumor tissue, green for CD3^+^ T cells and red for CD8^+^ T cells; scale bar = 40 μm. Data are presented as mean ± SD. Statistical analysis was carried out by means of one-way ANOVA. ∗*P* < 0.05; ∗∗*P* < 0.01; ∗∗∗*P* < 0.001; ∗∗∗∗*P* < 0.0001; ns represents not significant.

Microorganisms activate Toll-like receptors through lipopolysaccharide and dsDNA. Synergistically, manganese ions prompt cGAS to recognize dsDNA and form cGAMP, which enhances the binding ability of STING to cGAMP, thereby activating the cGAS–STING pathway. Herein, ultrasound-induced release of bacterial dsDNA was expected to co-activate immunization with Mn. Since the tumor immune microenvironment in breast cancer is extremely complex and involves many immunosuppressive cells such as tumor-associated macrophages (TAM), and immunosuppression is highly correlated with poor prognosis^42^^,^^43^, breast cancer was chosen as a model to validate synergistic immune enhancement *in vivo*. Specifically, PBS, Mn-MOF, 5 × 10^5^ CFU of ECN, and HA@Mn-MOF@E were injected intratumorally into mice with 4T1 tumors. The immune cells in tumors and lymph nodes were analyzed, followed by cell gating strategies (Supporting Information Fig. S14). Compared with the ECN and HA@Mn-MOF groups, the HA@Mn-MOF@E + US group showed a significant increase in the proportion of CD3^+^ T cells within the tumors (Fig. 6A), as well as a significant increase in the proportion of M1/M2 macrophages (Fig. 6B), suggesting that the tumors were in an immune-activated inflammatory state. In the lymph nodes, the proportions of IFN-*γ*^+^CD8^+^ effector T cells and CD86^+^ DCs in the HA@Mn-MOF@E group increased approximately two-fold compared with those of the HA@Mn-MOF group (Fig. 6C and D), indicating the activation of antigen-presenting cells. The expression of inflammatory factors, such as *Tnfa*, *Cd206*, and *Il6*, and the relative content of STING pathway-related genes, such as *Ifnb*, *Isg*, and *Ccl5*, were also determined by PCR in tumor tissues (Fig. 6E–J). In the HA@Mn-MOF@E + US group, inflammatory factors such as *Tnfa* and *Il6* were upregulated, while *Cd206*, a marker of M2 macrophages, was down-regulated. The expression level of STING pathway-related genes was much higher than that in the other groups, suggesting that HA@Mn-MOF@E activates inflammatory responses by activating the STING pathway in tumor tissues. The significant activation response of the STING pathway in the HA@Mn-MOF@E + US group may be due to the superposition of ultrasound-triggered release of dsDNA from the bacteria and the enhancement of dsDNA recognition by Mn^2+^ ions. In addition, CD3 and CD8 staining of tumor tissue sections of HA@Mn-MOF@E on Day 4 after administration showed an increased number of CD3^+^ and CD8^+^ T cells (Fig. 6K), further confirming the enhanced immune response. Thus, HA@Mn-MOF@E is capable of combining the dual immune activation effects of bacteria and Mn to enhance the inflammatory response at the tumor site and strengthen the immune response in the lymph nodes.

### 3.6 *In vivo* anti-tumor effect

**Figure 7:**
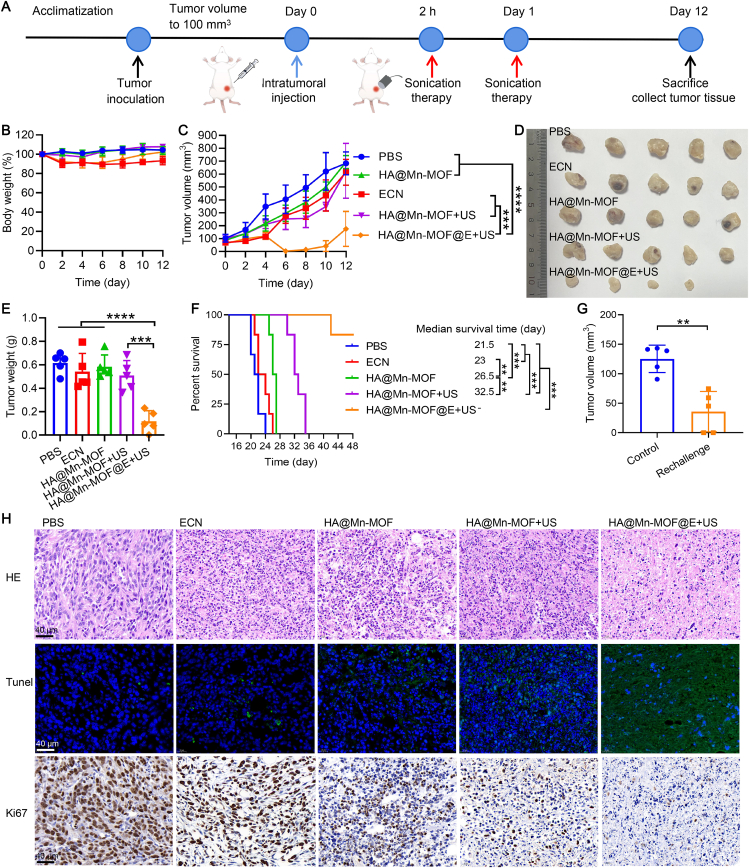
Bacterial sonosensitizer hybrid systems against orthotopic breast cancer. (A) Schedule of administration and US treatment; (B) curves of body weight (*n* = 5); (C) curves of tumor volume (*n* = 5); (D) tumor photographs on Day 12; (E) tumor weight (*n* = 5); (F) survival curves of intratumoral injection (*n* = 6); (G) tumor size in the contralateral breast of HA@Mn-MOF@E + US group mice on Day 8 after inoculation with 4T1 cells to rechallenge (*n* = 5); (H) staining image of H&E, TUNEL and Ki67 of tumor tissue sections; scale bar = 40 μm. Data are presented as mean ± SD. Statistical analysis was carried out by means of one-way ANOVA or Log-rank test. ∗∗*P* < 0.01; ∗∗∗*P* < 0.001; ∗∗∗∗*P* < 0.0001.

Breast cancer was previously selected for the local injection of HA@Mn-MOF@E, which has tumor tropism and uniform colonization, to enhance the efficacy of ultrasound-assisted immunotherapy *in situ*. Further antitumor effect of this bacterial hybrid system (*i*.*e*., HA@Mn-MOF@E) was validated using mice bearing *in situ* breast cancer. An orthotopic breast cancer model was established by injecting 1 × 10^6^ of 4T1 cells into the mammary glands of female BALB/c mice. When the tumor grew to 100 mm^3^, PBS, ECN, Mn-MOF, and HA@Mn-MOF@E containing the same number of bacteria (5 × 10^5^ CFU) or the same amount of Mn-MOF were injected into the tumor. Tumors in the US groups were treated with ultrasound at 1.5 W/cm^2^ for 5 min at 2 and 24 h after injection (Fig. 7A). After administration, there was a decrease in body weight of mice in the live microorganism groups (Fig. 7B), but ultrasound treatment led to a rebound in body weight, whereas body weight in the ECN administration group was maintained at a reduced level, suggesting that persistent infection of live microorganisms within tumors had a detrimental effect on mice. As shown in Fig. 7C, some tumors in the HA@Mn-MOF@E + US group completely disappeared on Day 6 after injection. Representative morphologic photographs of the mouse tumors on Day 8 are shown in Supporting Information Fig. S15, which demonstrated that the HA@Mn-MOF@E + US group had no apparent bulge and the tumor surface was darkened, suggesting a necrotic state internally of tumors. Moreover, on Day 12, the tumor tissues were photographed and weighed, and the tumor size of the HA@Mn-MOF@E + US group was found to be significantly shrunken compared to that of other groups (Fig. 7D and E). Monitoring the tumor volume in each mouse (Supporting Information Fig. S16) showed that although HA@Mn-MOF + US treatment without bacterial carrier delayed tumor growth, residual tumor tissue regrew rapidly, with no significant difference in tumor size on day 12 compared to the PBS group. Survival curves (Fig. 7F and Supporting Information Fig. S17) demonstrated that HA@Mn-MOF extended survival to 26.5 days, while the HA@Mn-MOF ultrasound-treated group extended survival to 32.5 days. Notably, even at 48 days, five mice in the HA@Mn-MOF@E + US group remained completely tumor-free without recurrence. On Day 40, five cured mice underwent rechallenge with 1 × 10^6^ of 4T1 cells inoculated into the contralateral mammary gland. By Day 8 post-rechallenge, tumor volumes (Fig. 7G) were smaller compared to the control group, and two mice remained recurrence-free (60% recurrence rate). After 3 days of treatment, hematoxylin and eosin (H&E) staining results (Fig. 7H) showed that the nuclei of the cells in the HA@Mn-MOF@E + US group were fragmented, and the nucleoplasm was separated. The terminal deoxynucleotidyl transferase dUTP nick end labeling (TUNEL) and Ki67 staining showed that, compared with the ECN group and the HA@Mn-MOF + US group, the tumor tissues in the HA@Mn-MOF@E + US group had increased apoptotic cells, and the Ki67-positive tumor cells had almost disappeared, implying that the tumors were in a necrotic state and had lost their proliferative ability. H&E staining of major tissue organs did not show any organ damage or toxicity of HA@Mn-MOF@E + US (Supporting Information Fig. S18). Besides, the tumors of the HA@Mn-MOF@E + US group also completely disappeared (Supporting Information Fig. S19A–S19C) *via* intravenous injection and showed no recurrence up to Day 45 (Fig. S19D), with significantly prolonged survival compared to other treatment groups. Thus, HA@Mn-MOF@E was verified to significantly inhibit the progression of orthotopic breast cancer by ultrasound-assisted-immunotherapy.

## 4 Conclusions

For the predicament of immune tolerance due to lack of specificity and long-term stimulation of live bacterial colonization, an sonosensitizer-modified bacterial delivery system was constructed to achieve robust activation of the antigen-presenting process by ultrasound stimulation. The Mn-containing porphyrin MOF was selected as a sonosensitizer to coat on the ECN bacterial surface by electrostatic interactions to construct bacterial sonosensitizer hybrid systems (*i*.*e*., HA@Mn-MOF@E). Ultrasound irradiation induced ECN death and tumor cell fragmentation to release antigen, and then the released Mn^2+^ ions and exogenous dsDNA effectively stimulated the cGAS-STING pathway to activate antigen-presenting cells. Both *in vitro* and *in vivo* immune effects confirmed the efficient activation of T cells, macrophages in the tumor, and DCs in the lymph nodes. The designed HA@Mn-MOF@E system, followed by ultrasound treatment, significantly inhibited the growth of orthotopic breast tumors. Such bacterial sonosensitizer hybrid systems represent a new paradigm for the use of live microorganisms for tumor colonization followed by ultrasound stimulation. Our study also proposes a complementary immune activation strategy for live microbe-mediated immunotherapy.

## Acknowledgments

We express our profound gratitude for the financial support furnished by the 10.13039/501100001809National Natural Science Foundation of China (Grant Nos. 82372098 and 82104073), and 10.13039/501100012166National Key Research and Development Program of China (2024YFA1212000).

## Author contributions

Haiyan Guo: Conceptualization, Methodology, Investigation, Writing-Original Draft. Yuhan Li: Validation, Methodology, Investigation, Formal analysis. Xue Chen: Investigation, Data Curation. Xiuru Ji: Investigation, Software. Zeyang Liu: Visualization. Hongjing Jiang: Project administration. Han Wang: Supervision. Dalong Ni: Conceptualization, Writing-Review and Editing, Funding acquisition.

## Conflicts of interest

All the authors declare no conflicts of interest.

## Appendix A Supporting information

The following is the Supporting Information to this article.
