# Supplementary material for: Development of bacterial sonosensitizer hybrid systems to enhance cancer sono-immunotherapy
Source: Acta Pharm Sin B. 2026 Mar 17;16(6):3920–33. doi: 10.1016/j.apsb.2026.03.024 (PMC13305165; doi:10.1016/j.apsb.2026.03.024)
Supplement: Multimedia component 1 [file mmc1.pdf]

Supporting Information for

Original article

Development of bacterial sonosensitizer hybrid systems to enhance cancer sono-immunotherapy

Haiyan Guo†, Yuhan Li†, Xue Chen, Xiuru Ji, Zeyang Liu, Hongjing Jiang, Han Wang, Dalong Ni*

Department of Orthopaedics, Shanghai Key Laboratory for Prevention and Treatment of Bone and Joint Diseases, Shanghai Institute of Traumatology and Orthopaedics, Ruijin Hospital, Shanghai Jiao Tong University School of Medicine, Shanghai 200025, China

Received 25 June 2025; received in revised form 18 September 2025; accepted 16 October 2025

*Corresponding author.

E-mail address: ndl12353@rjh.com.cn (Dalong Ni).

†These authors made equal contributions to this work.

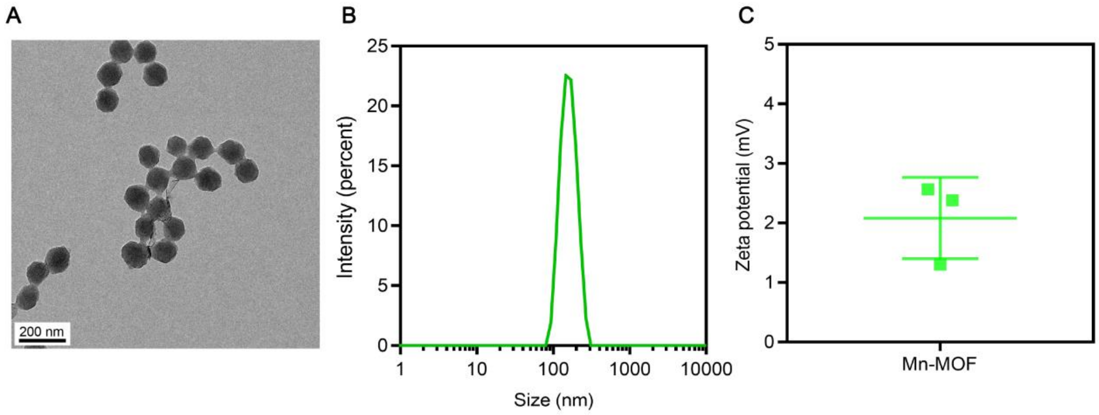

Figure S1. The characterization of Mn-MOF. TEM (A), particle size (B) and zeta potential (C) of Mn-MOF prepared by hydrothermal method ($n = 3$). Data are presented as mean ± SD.

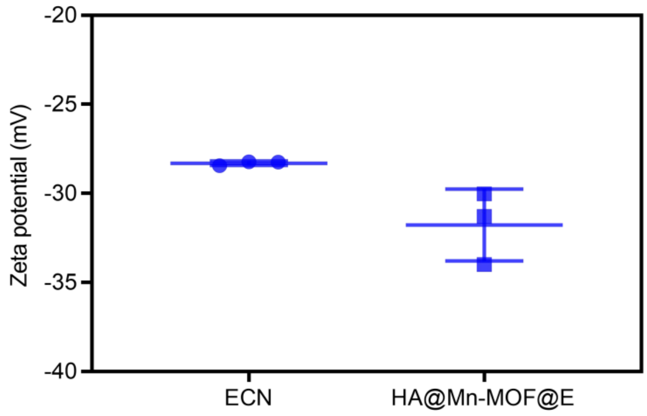

Figure S2. Zeta potentials of ECN and HA@Mn-MOF@E (*n* = 3). Data are presented as mean ± SD.

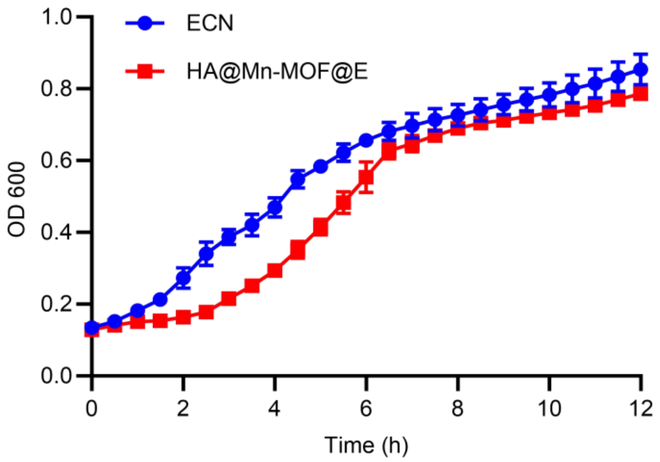

Figure S3. The growth curves of ECN and HA@Mn-MOF@E (*n* = 3). Data are presented as mean ± SD.

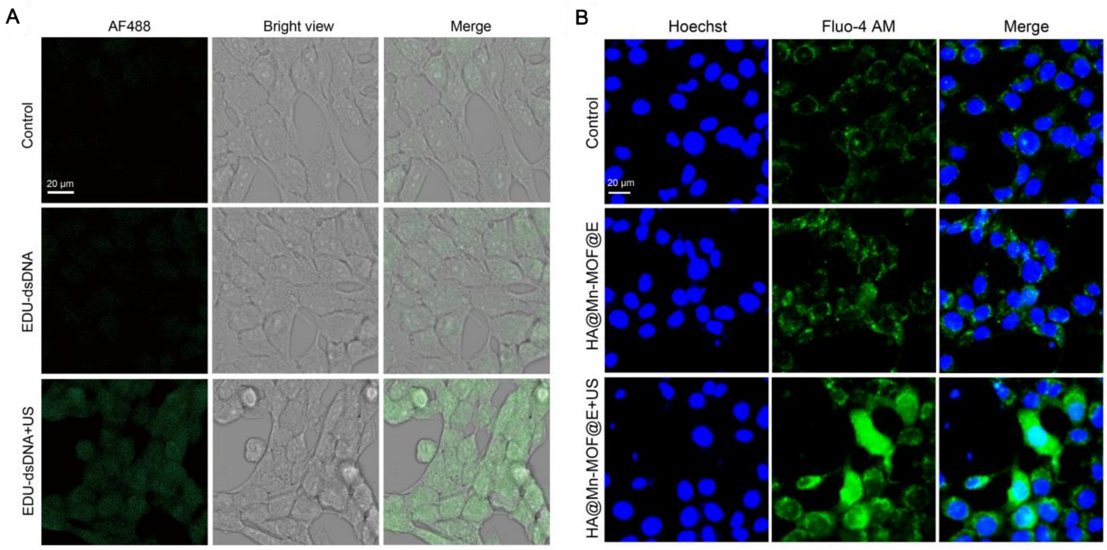

Figure S4. The cellular uptake effects of dsDNA and manganese ions. (A) Fluorescent staining images after co-incubation of the released EDU-dsDNA with 4T1 cells under transient ultrasonic treatment. (B) Mn ion staining images after co-incubation of sonicated HA@Mn-MOF@E with 4T1 cells for 1 h. Scale bar = 20 µm.

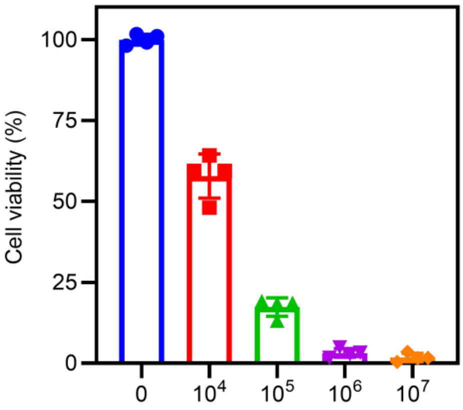

Figure S5. Cell viability after ultrasonic treatment with HA@Mn-MOF@E at different concentrations (n = 4). Data are presented as mean ± SD.

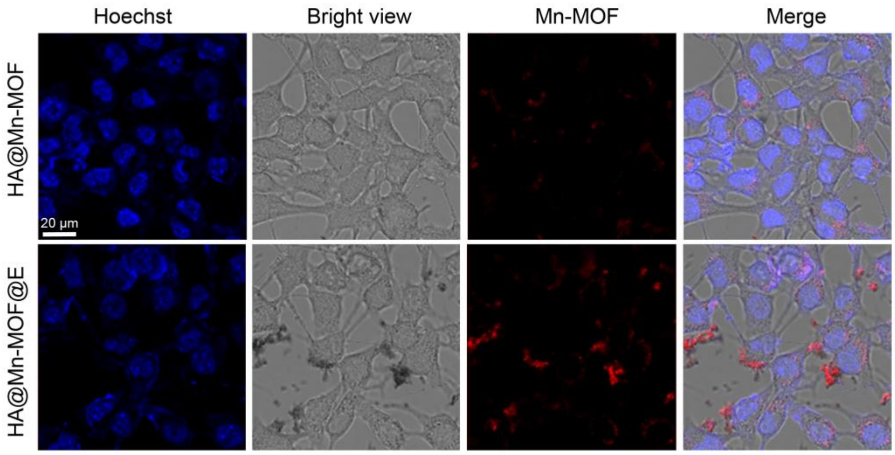

Figure S6. Confocal microscopy images of 4T1 cells co-incubated with HA@Mn-MOF or HA@Mn-MOF@E with the same ion concentration for 1 h; scale bar = 20 μm.

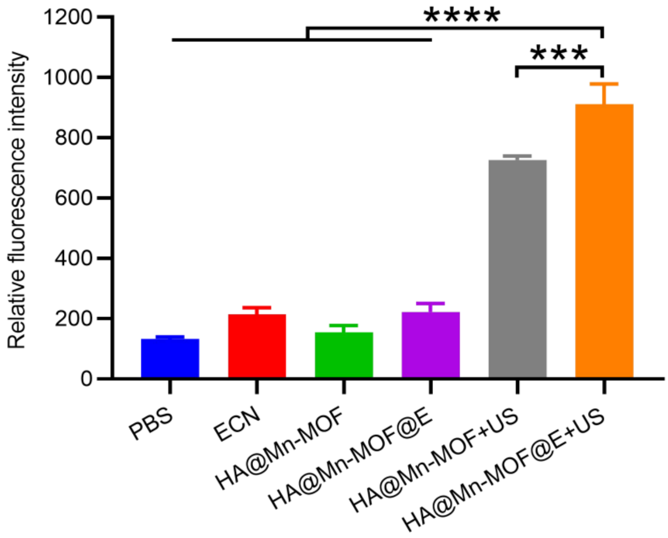

Figure S7. Sonodynamics-induced changes in singlet oxygen levels (n = 3). Data are presented as mean ± SD. ***$P < 0.001$; ****$P < 0.0001$.

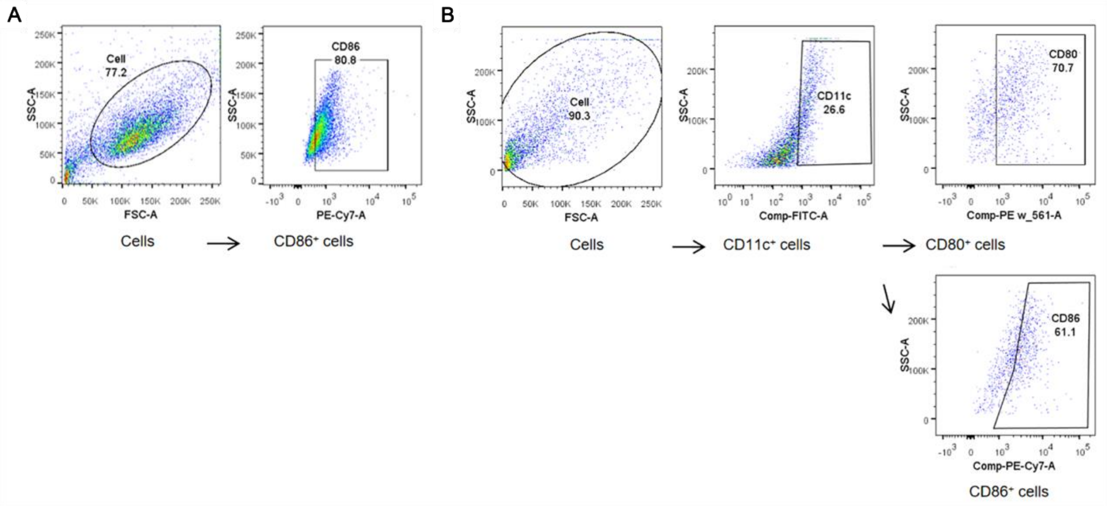

Figure S8. Gating strategies for macrophages (A) and DCs (B).

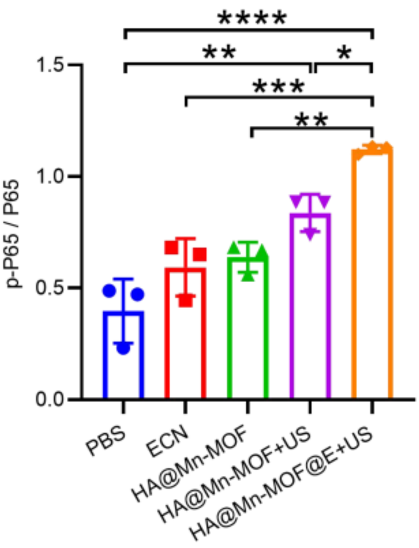

Figure S9. Quantitative data showing p-P65 protein levels in macrophages after 12 h co-incubation with each group (n = 3). Data are presented as mean ± SD. *P < 0.05; **P < 0.01; ***P < 0.001; ****P < 0.0001.

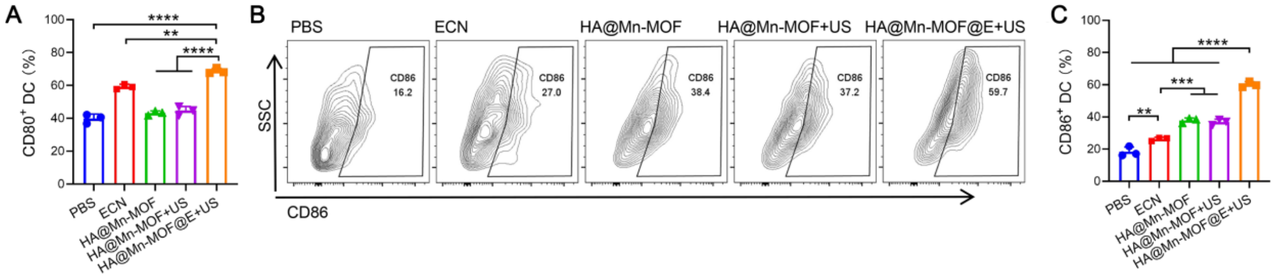

Figure S10. The quantitative data of $CD80^+$ DC (A); The representative (B) and quantitative data (C) of DC activation through co-incubation with PBS, ECN, HA@Mn-MOF, sonicated HA@Mn-MOF and sonicated HA@Mn-MOF@E (n = 3). Data are presented as mean ± SD. $^{**}P < 0.01$; $^{***}P < 0.001$; $^{****}P < 0.0001$.

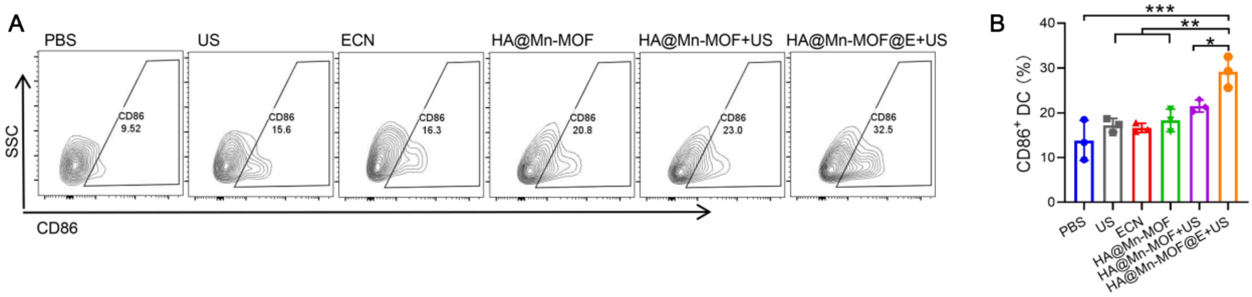

Figure S11. The representative (A) and quantitative data (B) of DC activation through co-incubation with the supernatant of treated tumor cells (n = 3). Data are presented as mean ± SD. $^{*}P < 0.05$; $^{**}P < 0.01$; $^{***}P < 0.001$.

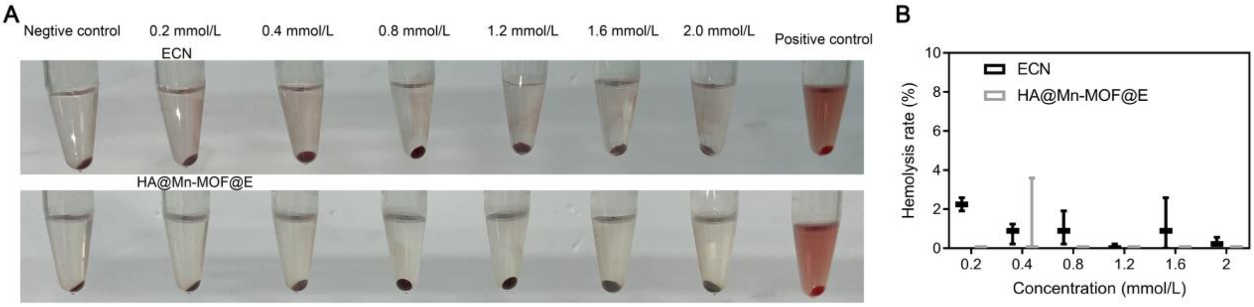

Figure S12. Hemolysis of ECN and HA@Mn-MOF@E at Mn-calibrated bacterial concentrations in mouse blood (n = 3). Data are presented as mean ± SD.

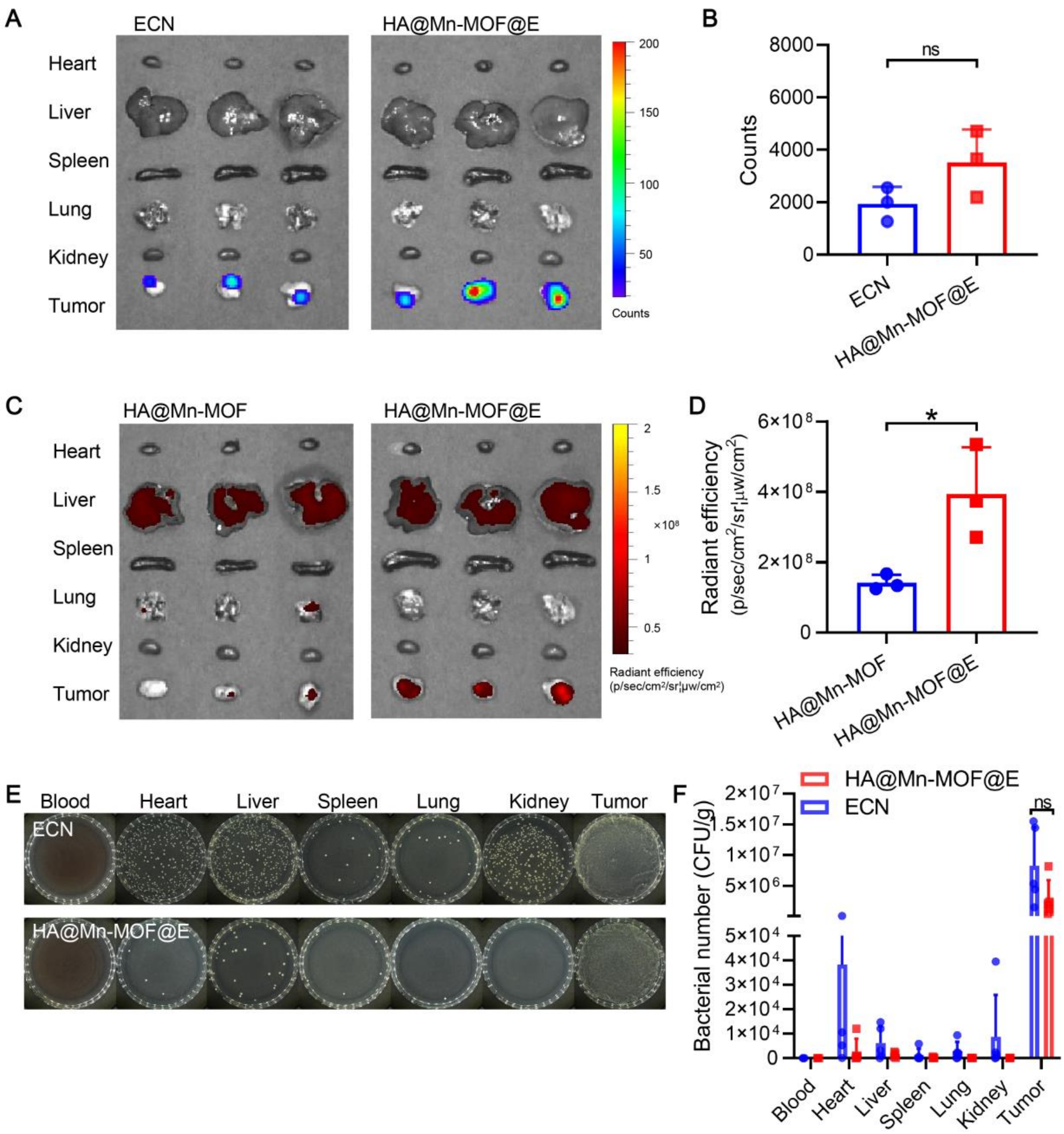

Figure S13. Targeted colonization ability at the tumor site. Luciferase-expressing ECN was modified with Mn-MOF and then intravenously injected into mice bearing 4T1 subcutaneous tumors at a dose of 5×10^5 CFU. (A) Bioluminescence imaging of major tissues at 72 h and (B) quantitative statistical results (n = 3). Meanwhile, the same amount of Mn-MOF was intravenously injected. (C) Fluorescence imaging (633 nm) from porphyrins in MOF at 72 h and (D) quantitative statistical results (n = 3). At 72 h after injection of ECN and HA@Mn-MOF@E, (E) plate photos and (F) quantitative statistical results of bacterial number in blood and major tissues (n = 5). Data are presented as mean ± SD. Statistical analysis was carried out by means of unpaired Student's t test. *$P < 0.05$; ns represents not significant.

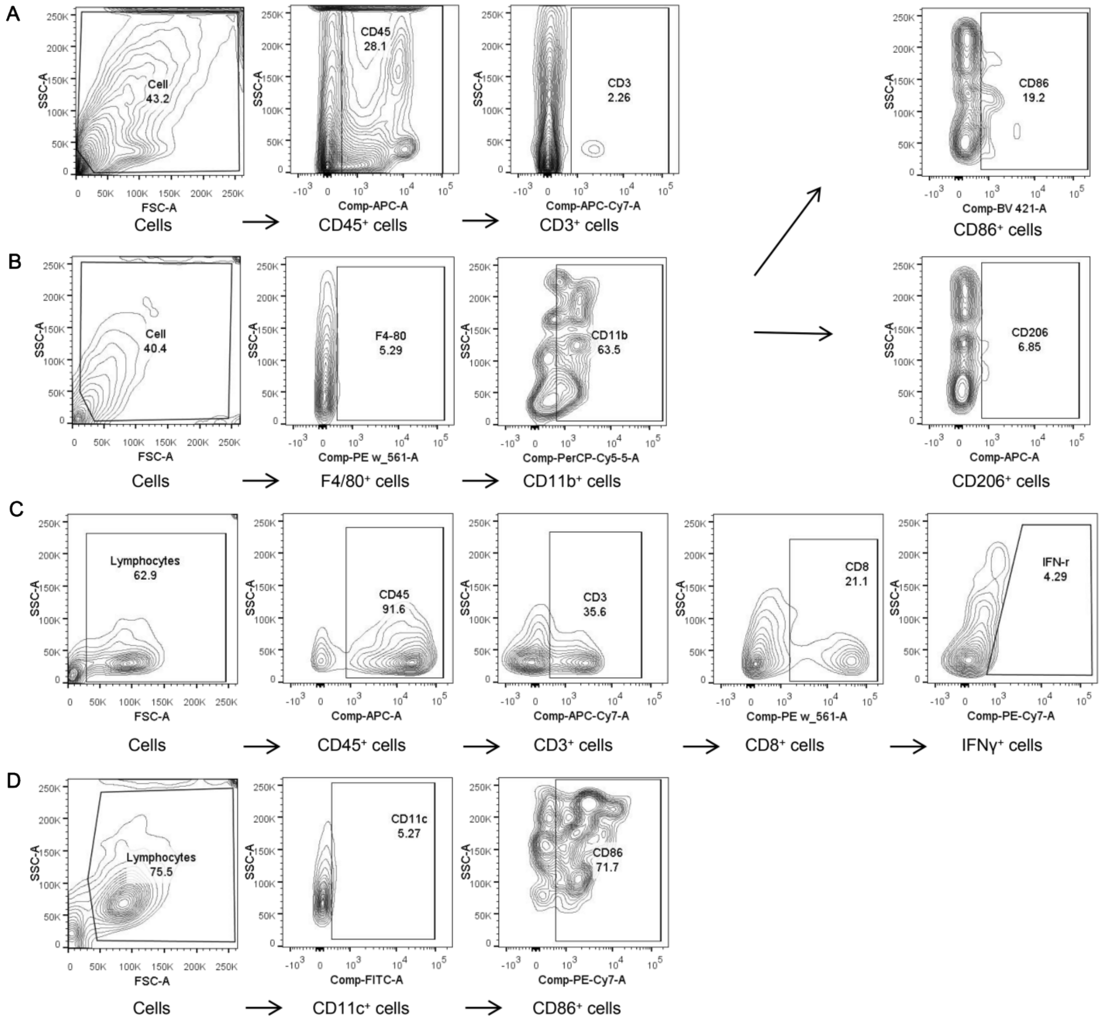

Figure S14. The gating strategies of flow cytometric analysis for $CD3^+$ T cells in tumor (A), M1 and M2 type of macrophages in tumors (B), IFN-γ^+ $CD8^+$ T cells in lymph node (C) and $CD86^+$ DCs in lymph node (D).

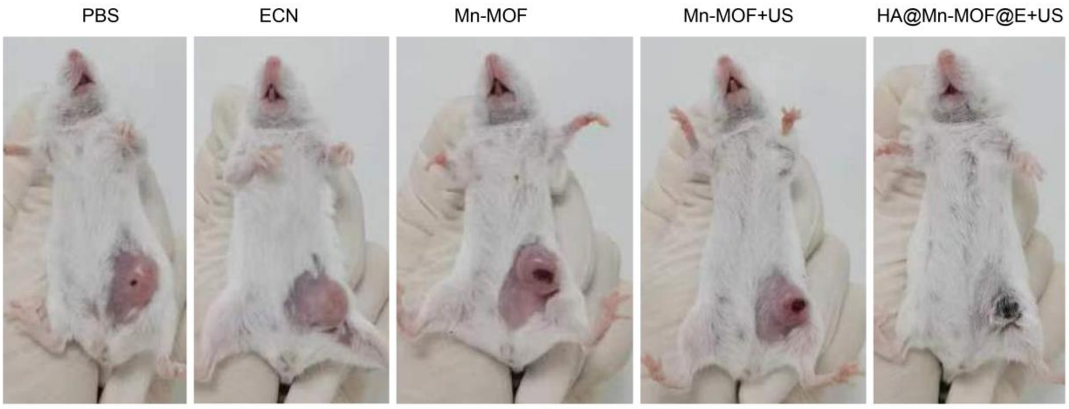

Figure S15. Representative photographs of tumor surface morphology on Day 8 after drug administration in mice with 4T1 *in situ* mammary tumors treated with PBS, ECN, HA@Mn-MOF, HA@Mn-MOF+US, and HA@Mn-MOF@E+US, respectively.

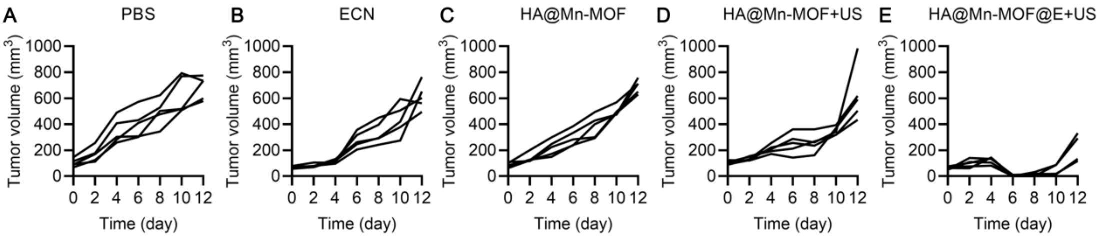

Figure S16. The curve of tumor volume of each mouse.

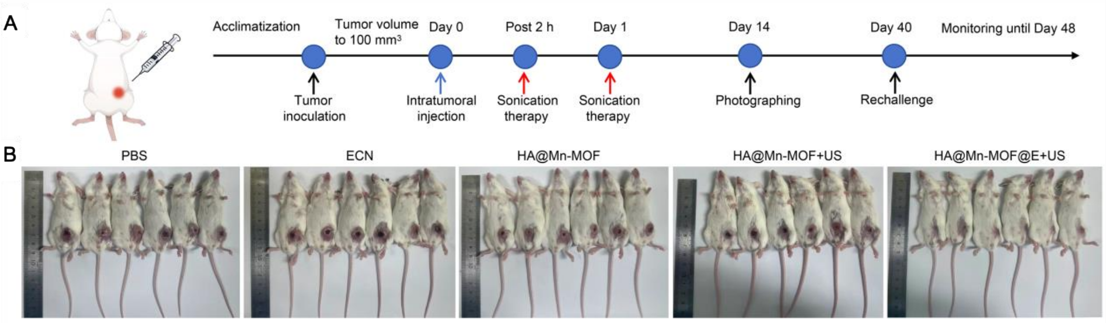

Figure S17. Intratumoral injection pharmacodynamics. (A) The timeline of treatment; (B) photographs of mice from each group on Day 14 post-intratumoral injection.

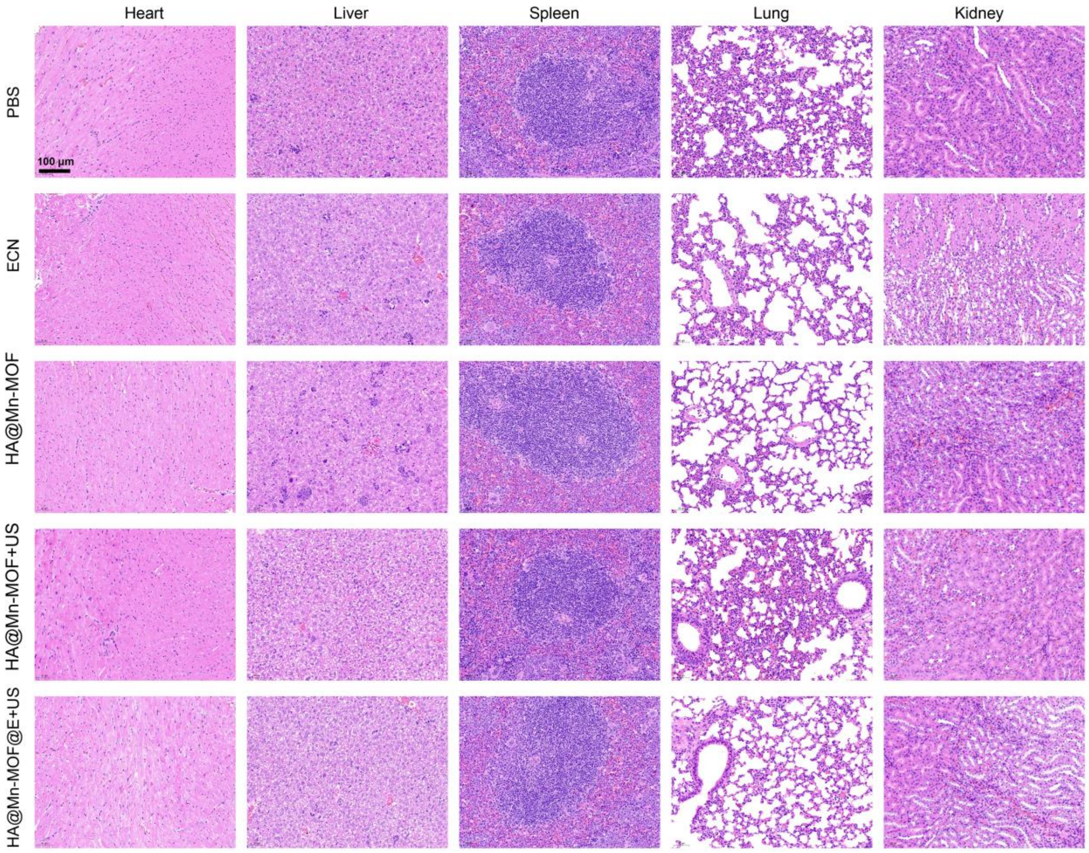

Figure S18. H&E staining of heart, liver, spleen, lung, and kidney of *in situ* 4T1 mice on Day 3 after intratumorally injection with PBS, ECN, HA@Mn-MOF, and HA@Mn-MOF or HA@Mn-MOF@E followed by US treatment, respectively.

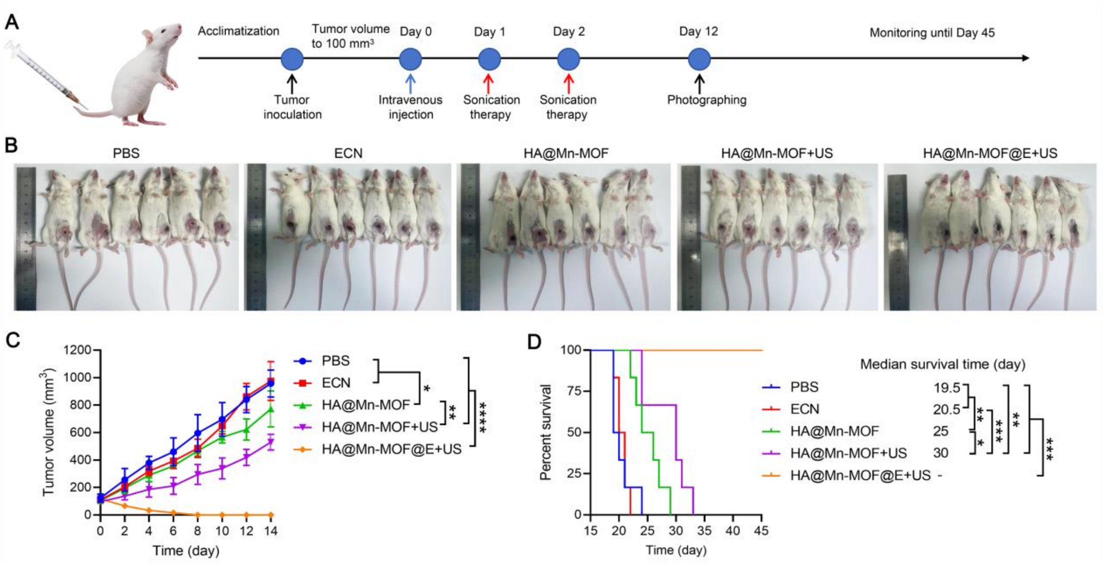

Figure S19. Efficacy of intravenous HA@Mn-MOF@E treatment for *in situ* mammary tumors in mice. (A) The timeline of treatment; (B) tumor photographs on 12 days after intravenous injection of PBS, ECN, HA@Mn-MOF, and HA@Mn-MOF@E in mice; (C) tumor volume change curve (n = 6); (D) survival curve (n = 6). Data are presented as mean ± SD. Statistical analysis was carried out by means of one-way ANOVA or Log-rank. *$P < 0.05$; **$P < 0.01$; ***$P < 0.001$; ****$P < 0.0001$.
